# Covert lie detection using keyboard dynamics

**DOI:** 10.1038/s41598-018-20462-6

**Published:** 2018-01-31

**Authors:** Merylin Monaro, Chiara Galante, Riccardo Spolaor, Qian Qian Li, Luciano Gamberini, Mauro Conti, Giuseppe Sartori

**Affiliations:** 1 PhD Program in Brain, Mind and Computer Science, University of Padova, Padova, 35121 Italy; 2 Department of General Psychology, University of Padova, Padova, 35131 Italy; 3 Human Inspired Technology Research Centre, University of Padova, Padova, 35121 Italy; 4 Department of Mathematics, University of Padova, Padova, 35121 Italy

## Abstract

Identifying the true identity of a subject in the absence of external verification criteria (documents, DNA, fingerprints, etc.) is an unresolved issue. Here, we report an experiment on the verification of fake identities, identified by means of their specific keystroke dynamics as analysed in their written response using a computer keyboard. Results indicate that keystroke analysis can distinguish liars from truth tellers with a high degree of accuracy - around 95% - thanks to the use of unexpected questions that efficiently facilitate the emergence of deception clues.

## Introduction

Presently, one of the major issues related to identity and security is represented by the numerous and current threats of terrorist attacks^1^. It is known that terrorists cross borders using false passports or claiming false identities^2^. For example, one of the suicide bombers involved in the Brussels train-station attack on March 22, 2016 was travelling through Europe using the identity of an Italian football player^3^. In these scenarios, security measures mainly consist of cross-checks on the data declared by the suspects or in the verification of biometric features, such as fingerprints, hand geometry, and retina scans^4^. Nevertheless, terrorists are mostly unknown: they are not listed in any database, and the security services do not have any information by which to recognise them.

False identities in online services represent another unresolved issue. They expose people to the risk of being attacked and manipulated^5,6^. Existing security systems for online authentication are now primarily based on passwords, while other biometric methods have been recently proposed^7^. These are based on human-computer interaction recording, such as systems for user authentication and identification via keystroke analysis or mouse dynamics^8^. In short, machines are trained to recognise the typical usage pattern of the keyboard/mouse of a specific user. However, these methods entail acquiring the writing or the mouse movement pattern of each user and storing it in a database queried upon every authentication.

Recently, Monaro *et al*. proposed a novel methodology which, using mouse dynamics in response to unexpected questions, identifies fakers of personal identities with a high degree of accuracy^9–11^. In brief, the authors developed a task in which they presented liars and truth tellers with questions related to their ID-card identities. Participants were asked to answer “yes” or “no” to questions by clicking the correct response on the computer screen with the mouse in accordance with the personal information on their ID card. Questions given to participants were of three types: control, expected and unexpected questions. The first set of questions was related to personal features about which no one could lie in a physical setting (e.g. “are you a female?”). Expected questions were questions about the information on the ID card (e.g. “were you born in 1990?”). Finally, unexpected questions were derived from the ID card information (e.g. “are you 26 years old?”). Unexpected questions may be retrieved directly by truth tellers, while liars would compute them on the fly, and this additional working-memory load shows itself in the increased number of errors and in mouse trajectories and kinematics.

However, the yes/no structure of the test requires preliminary crafting of the questions by the experimenter, and this may be problematic for the online application of the technique. In order to overcome such caveats, we will report here an experiment in which participants respond to similar questions entering their response in an edit box using the keyboard. In the present work, we asked unexpected questions of participants, but, innovatively with respect to Monaro *et al*.^11^, we recorded the subjects’ typing pattern on the keyboard (keystroke dynamics). The main advantage of relying on keystroke dynamics is that, in contrast to mouse tracking, it can also be adopted in situations in which it is not possible to formulate close-ended “yes or no” questions, such as the online context (e.g. a website subscription form).

In short, we will report data from an experiment that registered the keyboard dynamics when participants answered questions related to personal identity not requiring preliminary information about the examinee.

## Cognitive Mechanisms of Deception

In order to identify liars and, more generally, the production of deceptive responses, several authors have studied the cognitive mechanisms involved in deception. There is a broad consensus in the literature that producing a lie is cognitively more complex than truth telling. It requires the inhibition of the true response and its substitution with a lie which, in turn, should be not easily verified as such^12^. The major involvement of memory load is revealed by the increased number of errors and longer response times^13–15^. In a similar way, some authors have argued that the writing pattern of an individual (i.e. keystroke dynamics) may provide a clue to recognising deception. Keystroke dynamics refers to detailed timing information regarding human typing rhythm: it describes exactly when each key is pressed and released while a person is typing at a computer keyboard, a mobile phone or a touchscreen panel^16^. Typing pattern analysis is an implicit-behaviour measure insofar as the user is not aware of it during interaction with the device^17^. The keystroke features most commonly extracted are latencies, for example, how long a key is held down (dwell Time), or the time between the release of one key and the pressing of the next (flight time)^16^. Grimes *et al*.^18^ proposed a model to explain the relationship between deception and keystroke dynamics.

Few studies in the literature have applied keystroke dynamics to identify the production of false information. Moreover, all such studies have focussed on the identification of deception in online reviews^19^ or in chat interplay^20^. The results support the cognitive-load theory, confirming that deception is correlated with keystroke features. Up to now, however, no study in the literature has reported results on the identification of identity deception via keystroke pattern analysis.

To date, scientific lie-detection methods based on the cognitive mechanism of deception that may be used to unmask false identities are the autobiographical Implicit Association Test (aIAT)^21^ and the Concealed Information Test (CIT)^22^. It has been shown that CIT and aIAT spot liars with an accuracy of 90%. However, their practical application is limited, as they require the use of the to-be-tested true information. In other words, CIT and aIAT can detect which one between two information is true and which is false, while in the real cases only one information, the one that the examinee declared, is available. Finally, both CIT and aIAT perform overt lie detection.

Overt lie detection includes all techniques for which the examinee knowingly takes a lie-detection test. This category includes the polygraph, P300, fMRI, CIT, aIAT and others^23^. Covert lie detection refers to conditions under which the examinee is unaware that he or she is under the scrutiny of a scientifically based lie-detection technique. Such covert lie-detection techniques include thermal imaging lie detection^24^, the voice stress analyser^25^, mouse tracking^11^ and linguistic analysis^19,26^.

The technique that we present here falls under the category of covert lie-detection techniques. That is, the subject responded to questions by digitising, in an edit box, his or her responses as free text. There was no constraint or specific instruction to follow, for the response collection mimicked the typical online form. For research purposes, liars were instructed to respond with a fake identity, but, in a real setting, no hint is given to the subject that he or she is under the scrutiny of a credibility-assessment technique.

## Methods

The experiment aims to investigate whether there are differences in the writing patterns of a person who provides real personal information and a person who, on the contrary, provides intentionally false personal information. Half of participants were instructed to lie about their identity, while the remaining half entered their real information.

In our experiment, similar to the experimental design in Monaro *et al*.^9–11^, we used unexpected questions to increase the cognitive load (especially for liars). The use of unexpected questions has been shown to be effective in uncovering deception in investigative interviews^27^. In our experiment, expected questions are information typically reported on the ID card (e.g. month of birth), while unexpected questions are information that liars have to compute starting from the available expected information (e.g. the zodiac sign) and that truth tellers have, in contrast, readily available.

Given that lying taps further into working memory than truth telling, we expect keystroke dynamics to reflect this cognitive difference. We expect longer reaction times (RT) and more errors in liars relative to truth tellers, especially in response to unexpected questions, a type of question that requires a higher cognitive load. Moreover, in line with the model of Grimes *et al*.^18^, we expect to detect a greater variability in liars’ typing patterns and, conversely, minor deviations from average values in truth tellers’ typing patterns.

### Experiment

**Figure 1:**
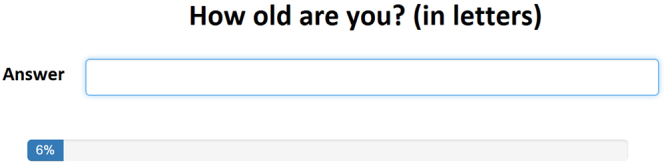
Example of the computer screen. Participant were instructed to respond writing in the edit box which was located below the presented sentence. The subject was instructed to finish the response pressing ENTER.

The experiment was conducted in the laboratories of the School of Psychology at Padua University using a single laptop ASUS F552WE-SX039 15.6” in order to avoid possible device-specific variation. The experiment was run from a site built using PHP, HTML and JavaScript. Recording of keystrokes and intervals was programmed using JavaScript. Through the website, we collected the responses of 60 individuals who completed the edit box presented below (see Fig. 1) with the appropriate autobiographical information. The experimental online task is accessible through this link: https://truthorlie.math.unipd.it/new/, and the task code is publicly available at this link: https://github.com/SPRITZ-Research-Group/Covert_lie_detection_using_keyboard_dynamics. Data were stored via MySQL Ver 14:14 Database. Finally, data were analysed using R^28^ for preliminary descriptive statistical analysis and WEKA^29^ for developing the machine-learning (ML) models trained to classify whether the collected response was that of a truth teller or that of a liar. Data are publicly available at this link: https://github.com/SPRITZ-Research-Group/Covert_lie_detection_using_keyboard_dynamics, as is the R code used for preliminary descriptive statistical analysis and the description of the WEKA procedure followed to obtain the ML models. For the sake of clarity, ML refers to the study and construction of algorithms that can learn information from a set of data (called a training set) and make predictions for a new set of data (called a test set). ML is now the basis for a large number of applications, such as the self-driving cars, speech recognition (e.g. Siri), recommender systems, etc. It enables the training of one or more algorithms to predict outcomes without being explicitly programmed and only uses the information learned from the training set. Usually, ML models outperform traditional statistical models.

### Participants

A first sample of 40 participants—12 males and 28 females—was recruited (average age = 23 years [sd = 1.9], average education level = 17 years [sd = 1.8]). These 40 participants were used to preliminarily build an ML classification model (training set). When the model was built, a new group of 20 participants—6 males and 14 females—was recruited to test the classification model (test set: average age = 22 years [sd = 1.7], average education level = 16 years [sd = 1.6]).

All participants signed an informed-consent agreement. A photo of each participant’s face was taken and pasted on a standard Italian ID card together with the personal information of the participant. Debriefing at the end of the experiment was carried out.

The experimental procedure was approved by the ethics committee for psychological research in the Padova University Psychology Department. The experiment was performed in accordance with relevant guidelines and regulations.

### Experimental Procedure and Stimuli

The experimental procedure is similar to the one adopted by Monaro *et al*.^9–11^. We had 20 participants to answer truthfully, while the others were instructed to lie about their identity according to a false autobiographical profile which was presented on the fake ID card displaying the participant’s real photo and false personal information. After the learning phase, participants were required to correctly recall the information presented on the ID card twice; they performed a mental-arithmetic task in between. This multistep procedure ensured the investigator that liars actually learned their assigned false personal information.

For both experimental groups, the task required answering 18 open-ended questions related to identity. Table 1 reports the list of presented questions. The 18 sentences were displayed on the central area of the screen. Figure 1 shows an example of the presentation screen and the location of the edit box filled out by the participants. At the end of the responses, participants were instructed to press the ENTER key. A bar in the lower part of the computer screen indicated the percentage of the test completed at any given moment.

**Table 1:** List of the 18 questions presented to participants divided by type (control, expected and unexpected questions).

Question type	Question text
Control	What is your gender? What is the colour of your skin? What is the colour of your hair? What is your nationality?
Expected	What is your name? What is your last name? In which year were you born? In which month were you born? In which city were you born? In which city do you live? What is your home address? What is your e-mail address?
Unexpected	How old are you? (in letters) Which is your zodiac? In which region were you born? In which province were you born? In which region do you live? Which is the capital town of your residence region?

Before starting the experiment, participants completed a warm-up block consisting of three questions. Data collected from the warm-up block were not further analysed.

The 18 questions, randomly presented to subjects, belonged to the following categories:

- Control questions (n = 4): these included personal and physical information that was not possible to hide from the examiner in the face-to-face experimental setting (e.g. “are you a male?”). All participants, including liars, responded to these questions truthfully.
- Expected questions (n = 8): these were questions targeting information that appeared on the ID card. In the case of liars, the answers to these questions about their fake IDs were learned in the preliminary part of the experiment. Truth tellers responded truthfully to these questions while liars were required to lie and provide the information found on their fake ID.
- Unexpected questions (n = 6): unexpected questions were based on information strictly related to identity but not explicitly rehearsed in the preliminary phase by truth tellers or by liars. Truth tellers responded to these questions referring to their real identity, while liars responded referring to their assigned fake identity.

The control, expected and unexpected questions shown to participants during the experiment are reported in Table 1.

Typical response length ranged between 1 and 4 words. The total duration of the task was about 10–15 minutes.

### Behavioural Measures

For each response, the following data were collected and stored for analysis:

- Number of errors: this refers to the total number of errors committed by the person in answering the 18 questions. Specifically, error means the number of fields for which incorrect information was entered. Errors were calculated by checking each response given by the subject against the conceptually correct information. We only considered conceptual errors for three reasons. First, other types of errors, such as typing errors, were rarely detected because of the low number of words required by the responses. Second, when encountered, such errors were minimal, thereby not affecting the conceptual correctness of the answer. Third, an indirect measure of typing errors was already given by the use frequency of special characters, such as Del and Canc keys. An example reported as a conceptual error was the answer “Capricorn” when the corresponding date of birth of the learned profile was the December 2, 1988. Another example based on the same date of birth would be the answer “22” to a question related to age. Most conceptual errors were detected in unexpected questions, when the subjects, truth tellers or liars, were required to compute the unexpected information asked from the known data written on their own profile (real or assigned/fake).
- Prompted-firstdigit: this refers to the interval between the onset of the sentence on the computer screen and the first key pressed. This index was subsequently adjusted using a readability index for the Italian language (GULPEASE Index) in order to refine the reaction time by weighting the latency of the response to the question for the difficulty of reading the question^30^. GULPEASE is an index which takes into account the length of the sentence read.
- Prompted-enter: this refers to the total time from the stimulus onset to ENTER (pressed at the end of the response).
- Firstdigit-enter: this refers to the time between the first key and ENTER.
- Time before enter key: this refers to the time between last key and ENTER.
- Answer length: this refers to the number of characters of the response.
- Writing time: this refers to the average typing speed (firstdigit-enter/number of characters).
- Down time: this refers to the timestamp for pressing each key.
- Up time: this refers to the timestamp for releasing each key.
- Up and down time: this refers to the sum of down time and up time for each key.
- Press time: this refers to the duration between each key down and each key up.
- Flight time: this refers to the interleaving time between each key up and the next key down.
- Di-graphs: these refer to the sum of up time, down time or up and down time for two consecutive keys.
- Tri-graphs: these refer to the sum of up time, down time or up and down time for three consecutive keys.
- Frequency of use for special characters: this refers to the total number of key pressing for Shift, Del and Canc, Space and Arrows characters.

We calculated a total of 62 attributes for each subject, averaging each variable over the 18 responses given by each subject. The complete list of the 62 attributes is reported in Supplementary Table S1. Average, maximum, minimum, median, standard deviation and variance were calculated and statistically analysed for a preliminary identification of significant differences between truth tellers and liars.

The data set generated and analysed in the current study is available from the corresponding author upon reasonable request.

### Data availability statement

The datasets generated during and/or analysed during the current study are available in the “Covert_lie_detection_using_keyboard_dynamics” repository, https://github.com/SPRITZ-Research-Group/Covert_lie_detection_using_keyboard_dynamics.

## Results

### Statistical Analysis

A first analysis was carried out by examining the statistical differences in the collected data for truth tellers and liars through independent t-test. We ran a Welch’s t-test (included in the R software ‘lsr’ package), which adjusts the number of degrees of freedom when the variances are not assumed to be equal^31^. To avoid the multiple-testing problem, the Bonferroni correction was applied and the p-value was set to 0.0008. Furthermore, we calculated the Cohen’s d effect size. Results are presented in Table 2. This analysis revealed that liars make more errors, are slower in initiating their responses and are slower in total response time (from the stimulus onset to the confirmation of the response as characterised by pressing ENTER). No other variables collected reached a statistically significant value in the t-test.

**Table 2:** Table reports the t-value and p-value of the 4 attributes which revealed a statistically significant difference between the two groups (truthtellers vs liars), considering a significance level of p < 0.0008. Effect-size Cohen’s d is also reported.

Feature	*t*-test (*t*-value and *p*-value)	Effect-size (Cohen’s *d*)
Errors	*t*_(21)_ = −10.57, *p* < 8e-4	*d* = 3.34 (large)
Prompted-firstdigit	*t*_(31)_ = −6.34, *p* < 8e-4	*d* = 2.00 (large)
Prompted-firstdigit adjusted GULPEASE	*t*_(30)_ = −6.48, *p* < 8e-4	*d* = 2.05 (large)
Prompted-enter	*t*_(26)_ = −5.46, *p* < 8e-4	*d* = 1.73 (large)

We analysed the error rate separately for control, expected and unexpected stimuli. The analysis yielded the results reported in Table 3: the error rate is similar when responding to control and expected questions. In contrast, when responding to unexpected questions, liars produced 27 times more errors than truth tellers.

**Table 3:** Error rate to control, expected and unexpected question for liars and truthtellers.

Question type	Truthtellers	Liars
Control	0/80	0/80
Expected	0/160	3/160
Unexpected	3/120	81/120

### Feature Selection

It has been suggested that classifier accuracy is enhanced by selecting a subset of predictors which have maximum correlation with the dependent variable and minimal intercorrelation between features^32^. On the basis of these criteria, in a first-features selection step, we selected the predictors that show maximum correlation with the dependent variable. These predictors were as follows: number of errors (r_pb_ = 0.85), prompted-firstdigit adjusted for the GULPEASE index (r_pb_ = 0.71), prompted-firstdigit (r_pb_ = 0.70), prompted-enter (r_pb_ = 0.65), firstdigit-enter (r_pb_ = 0.46), writing time (r_pb_ = 0.50) and time before enter key down (r_pb_ = 0.43). In a second step, we looked at the intercorrelation between these seven features. Two of the seven predictors (prompted-firstdigit and prompted-enter) showed a very high correlation value, respectively, with prompted-firstdigit adjusted GULPEASE (r_pb_ = 0.99) and firstdigit-enter (r_pb_ = 0.89). Hence, to avoid redundancy, these features have been excluded. The five final attributes of the feature selection considered for the purposes of classification are described, as are their correlations, in Table 4. The entire correlation matrix between features and dependent variable can be found in the repository: https://github.com/SPRITZ-Research-Group/Covert_lie_detection_using_keyboard_dynamics.

**Table 4:** This table reports the correlation matrix among the five final predictor.

Feature	Number of errors	Prompted-firstdigit adjusted GULPEASE	Firstdigit-enter	Writing time	Time key before enter down	Feature
Number of errors	1.00	0.51	0.25	0.46	0.44	1.00
Prompted-firstdigit adjusted GULPEASE	0.51	1.00	0.66	0.60	0.54	0.51
Firstdigit-enter	0.25	0.66	1.00	0.67	0.52	0.25
Writing time	0.46	0.60	0.67	1.00	0.67	0.46
Time key before enter down	0.44	0.54	0.52	0.67	1.00	0.44

### Classifier Performance

Classifiers were run in WEKA, an ML software^29^. Four different classifiers were trained via a 10-fold cross-validation procedure using data from the first 40 participants as a training set^33–37^. We selected four classifiers that differ based on their assumptions. Random forest operates by constructing a multitude of decision trees^33^. Logistic regression measures the relationship between the categorical dependent variable and one or more independent variables by estimating probabilities using a logistic function^34^. Support vector machine (SVM) is a non-probabilistic binary linear classifier which maps the space, so the examples of the separate categories are divided by a clear gap that is as wide as possible^35,36^. Logistic model tree (LMT) combines logistic regression and decision-tree learning^37^.

Finally, to evaluate the generalisation of the results for completely new data, models were tested on 20 new participants not previously used in the learning phase. Accuracies obtained by the classifiers during training and testing are reported in Table 5. In order to highlight the relative importance of predictors in classification accuracy, we eliminated the predictors one by one and recalculated classification accuracy. This analysis yielded the following results:

- Errors: errors are a key attribute, as the results indicated that eliminating errors from predictors led to a decrease in classification accuracy around 80% for the cross-validation and around 68% for the test.
- Prompted-firstdigit: when this predictor is eliminated with its related variables (such as prompted-firstdigit and prompted-enter), the overall accuracy remains substantially high (around 90% for training and around 95% in the test). Furthermore, these results are similar for different classifiers.
- Firstdigit-enter, writing time and time before enter key down: when eliminating the firstdigit-enter variable using the predictors, the accuracy remains high (around 94.5% for the cross-validation and around 92.5% for the test). The same occurs when removing the writing time and time key before enter down.

**Table 5:** The table reports the percentage of accuracy obtained on the training set using a 10-fold cross-validation procedure and in the test set (20 new participants) for four different machine learning classifiers. In addition to accuracies, the table reports the weight average of True Positive Rate (TP Rate), False Positive Rate (FP Rate), Precision value, Recall value, F-Measure, Receiver Operating Characteristics (ROC) Area value and Precision-Recall Curve (PRC) Area value.

Classifier	Accuracy	TP Rate	FP Rate	Precision	Recall	F-Measure	ROC Area	PRC Area
**10-fold cross-validation**								
Logistic	90%	0.900	0.100	0.904	0.900	0.900	0.959	0.948
SVM (SMO)	95%	0.950	0.050	0.950	0.950	0.950	0.950	0.928
LMT	97.5%	0.975	0.025	0.976	0.975	0.975	1.000	1.000
Random Forest	92.5%	0.925	0.075	0.926	0.925	0.925	0.972	0.972
**Test**								
Logistic	100%	1.000	0.000	1.000	1.000	1.000	1.000	1.000
SVM (SMO)	90%	0.900	0.100	0.917	0.900	0.899	0.900	0.867
LMT	90%	0.900	0.100	0.917	0.900	0.899	1.000	1.000
Random Forest	95%	0.950	0.050	0.955	0.950	0.950	1.000	1.000

The accuracy obtained for each classifier and all classification metrics are reported, in greater detail, in Supplementary Table S2. In short, errors are the single most important predictor in identifying a liar for this ID test. Furthermore, the variables related to response latency (prompted-firstdigit adjusted GULPEASE), the writing time (firstdigit-enter and writing time) and the interval between the last key press and the confirmation of the response (time before enter key down) also contributed significantly to the identification of liars.

All these analyses were conducted taking into account the responses to all three types of questions (control, expected and unexpected). We specifically analysed control questions separately as both liars and truth tellers were required to respond truthfully to control questions. All classifiers yielded a classification around chance level for this type of question (47.5% for cross-validation and 50% for the test; classifier accuracies and classification metrics are reported in Supplementary Table S3), and this result indicates that responses to control questions between the two groups were virtually indistinguishable.

### Analysis of Normalised Predictors

One could argue that keyboard dynamics are modulated by a number of different variables such as age, cultural level and typing skills. Hence, the analyses reported above were conducted on raw data using two groups of subjects similar in age, cultural level and typing skills. In order to render the results generalisable, it would be interesting to see whether similar results hold not only for raw data but also for normalised predictors. To overcome this limitation, we ran the classification models again using only normalised indices, less influenced by inter-individual and environmental variables. These indices were:

- Average number of errors (number of errors/total number of questions)
- Writing time (firstdigit-enter/answer length)
- Prompted-firstdigit – prompted-enter (prompted-firstdigit minus prompted-enter)
- Writing time/prompted-firstdigit – prompted-enter [writing time/(prompted-firstdigit minus prompted-enter)]

Results from the five classifiers using the normalised predictors are reported in Table 6. In short, the high degree of accuracy in classifying truth tellers and liars is also confirmed for normalised predictors.

**Table 6:** The table reports the accuracies obtained from five different machine learning classifiers in the 10-fold cross-validation and in test set, using only normalized measures as predictors. In addition to accuracies, the table reports the weight average of True Positive Rate (TP Rate), False Positive Rate (FP Rate), Precision value, Recall value, F-Measure, Receiver Operating Characteristics (ROC) Area value and Precision-Recall Curve (PRC) Area value.

Classifier	Accuracy	TP Rate	FP Rate	Precision	Recall	F-Measure	ROC Area	PRC Area
**10-fold cross-validation**								
Logistic	90%	0.900	0.100	0.900	0.900	0.900	0.946	0.912
SVM (SMO)	92.5%	0.925	0.075	0.935	0.925	0.925	0.925	0.897
LMT	90%	0.900	0.100	0.917	0.900	0.899	0.985	0.986
Random Forest	95%	0.950	0.050	0.950	0.950	0.950	0.966	0.961
**Test**								
Logistic	100%	1.000	0.000	1.000	1.000	1.000	1.000	1.000
SVM (SMO)	90%	0.900	0.100	0.917	0.900	0.899	0.900	0.867
LMT	90%	0.900	0.100	0.917	0.900	0.899	1.000	1.000
Random Forest	100%	1.000	0.000	1.000	1.000	1.000	1.000	1.000

### Countermeasures and Alternative Efficient Models

Resistance to countermeasures is a central issue for all available lie-detection techniques. We did not directly test resistance to countermeasures in the present paper, but a number of reasons indicate that coaching subjects could be difficult, particularly as pertains to:

1. Errors to unexpected questions are diagnostic of lying, and the subjects should respond without errors in order to cheat the test, However, this seems impossible as subjects are already performing at their maximum level. There are no easy countermeasures to the number of errors; therefore, countermeasures are limited by keystroke dynamics.
2. Parameters used to encode keystroke dynamics and correlate with the dependent variable are high in number, and only some were used in building the original model. It is unlikely that the cheater succeeds in implementing countermeasures that simultaneously remain under voluntary control all possible efficient predictors.

To highlight these points, we have tested a new model that uses the following as predictors: (rpb = 0.85), prompted-firstdigit (rpb = 0.70), prompted-enter (rpb = 0.65), time before enter key flight (rpb = 0.43) and di-graph down time average (rpb = 0.38) (note that the predictors used in the original analysis reported in the paper included errors, prompted-firstdigit adjusted GULPEASE, firstdigit-enter, writing time and time before enter key down). Results for the new set of predictors for the sample of 40 participants are as follows (results with 10-fold cross-validation): Random Forest = 90%, Logistics = 92.5%, SVM = 95% and LMT = 97.5%. Results for the 20 participants of the validation sample were as follows: Random Forest = 90%, Logistics = 100%, SVM = 90% and LMT = 90%. Classification metrics are reported in Supplementary Table S4. These results clearly show that there are other sets of predictors (different from those originally reported in the paper) that can be used to efficiently classify the participants and that it is hard to countermeasures to control the entire set of efficient predictors.

### Classification of Liars Using Only Data from Truth Tellers

While liars were instructed to lie about their identity, truth tellers were instructed to respond freely without any specific instructions. Under this view, liars are responding in an anomalous way with respect to truth tellers. Normally, in a real situation, the majority of the subjects report true identities; only a few provide false information and show an anomalous pattern of response. In order to evaluate whether liars may still be identified based on their anomalous response style, we have applied an ML technique called anomaly detection^38^. Anomalies are data that have different patterns relative to normal instances. The detection of anomalies provides significant information and has applications in many fields. For example, the detection of anomalies is used in credit-card transactions, astronomical images or nuclear-plant functioning. Anomaly-detection techniques classify subjects after a training limited to the most frequent group, in our experiment the truth tellers^39^. At prediction, new instances with unknown class labels can either belong to the target class (the class learned during training, i.e. truth tellers) or to a new class that was not available during training (in our case, the liars). This type of learning problem is known as one-class classification. Following this logic, we tested whether a one-class classifier^38^ can classify liars satisfactorily even if the model is trained only using data from truth tellers. This ML algorithm was trained using logistic regression on the data of the 20 original truth tellers and tested on the new group of 20 participants (10 liars and 10 truth tellers). The one-class algorithm correctly classified 85% of the instances; specifically, it correctly classified 70% of the truth tellers as the target and 100% of the liars as the outlier (classification metrics are reported in Supplementary Table S5). When we run the test on a group of 30 liars and 10 truth tellers, results are 29/30 liars correctly classified and 7/10 truth tellers correctly classified. These results indicated that the classifier trained only on truth tellers can identify liars with high-level accuracy.

### Online Experiment

To further evaluate the model, a second experiment was conducted, with participants recruited via the Web. The procedure used in this experiment was the same as the one previously described and only minor adaptations for online administration. The experimental task for the truth-teller condition is available at: https://truthorlie.math.unipd.it/new-online/index1.php. The experimental task for liars is available at: https://truthorlie.math.unipd.it/new-online/index.php.

Participants were recruited through a mailing list of students and alumni. Participants were randomly assigned to the truth-teller or liar condition. Two hundred ninety-seven subjects started the experiment. Participants who did not satisfy the recruitment criteria were excluded from further analysis. In more detail we excluded: participants who did not respond to all stimuli (n = 55) or who completed the test using a smartphone or a tablet (n = 31); participants who did not speak Italian as first language (n = 3), to exclude the possibility that the response time was influenced by a poor knowledge of the language; participants who completed the experiment with the clear intention to sabotage it (n = 1); participants who took the task more than one time (n = 15); participants for whom the system did not record keystroke up time (n = 41).

After filtering the participants using these criteria, 151 participants (86 liars and 65 truth tellers) were used for the final analysis. It should be noted that the dropout rate was around 50%. Given that we recruited subjects among trusted participants and that comparable online lie detection experiments have reported a dropout rate around 30%^22^, this figure (50%) could look somewhat high. However, in this study the 24% of participants were excluded because of non-compliance of the technical instructions that were given in the recruiting email (they were instructed about avoiding to use smartphones or tablet or using the non-supported browser Explorer given that it was not recording the up-times). One may presume that people who lack of motivation do not focus on all the instruction details before clicking the test link, increasing the rate of participants to be excluded.

The final group of 151 participants consisted of 41 males and 110 females (average age = 41 years, sd = 14.1; average education = 19 years, sd = 3.4). Data are publicly available at the following link: https://github.com/SPRITZ-Research-Group/Covert_lie_detection_using_keyboard_dynamics.

Data from the new 151 online-recruited participants were used to evaluate the models built with the original sample of 40 participants. The features entered in the models were those reported in Table 4. Results from the four ML classifiers are reported in Table 7. As the table demonstrates, the classification performance averaged over the four classifiers was 89%.

**Table 7:** The table reports the percentage of accuracy obtained in a test set of 151 participants (86 liars and 65 truthtellers) recruited online. In addition to accuracies, the table reports the weight average of True Positive Rate (TP Rate), False Positive Rate (FP Rate), Precision value, Recall value, F-Measure, Receiver Operating Characteristics (ROC) Area value and Precision-Recall Curve (PRC) Area value.

Classifier	Accuracy	TP Rate	FP Rate	Precision	Recall	F-Measure	ROC Area	PRC Area
**Test on 151 online-recruited participants**								
Logistic	86.1%	0.861	0.135	0.864	0.861	0.861	0.930	0.911
SVM (SMO)	88.7%	0.887	0.093	0.902	0.887	0.888	0.897	0.857
LMT	90.1%	0.901	0.086	0.908	0.901	0.901	0.959	0.953
Random Forest	90.7%	0.907	0.078	0.916	0.907	0.908	0.980	0.977

A second model was evaluated using the alternative set of predictors mentioned above (errors, prompted-firstdigit, prompted-enter, time before enter key flight and di-graph down-time average). The model was built on the original sample of 40 participants and tested on the 151 online-recruited participants. Results for the test set are as follows: Random Forest = 90%, Logistic = 90.1%, SVM = 90.1% and LMT = 90.7%. Classification metrics are reported in Supplementary Table S6.

Finally, running a 10-fold cross-validation on the 151 online-recruited participants (features: errors, prompted-firstdigit adjusted Gulpease, firstdigit-enter, writing time, time key before enter down) the accuracy of the four classifiers is in the range of 92–94%.

These results confirm that the proposed technique can spot liars with high-level accuracy even when administered online.

## Discussion

The novelty of this work is in the mean that has been apply to spot liars, which extends the possible applications of the unexpected-questions technique proposed by Monaro *et al*. in previous research^9–11^. Monaro *et al*.^11^ presented a technique based on unexpected questions that was effective in uncovering liars (specifically lying about personal identity). While the authors required participants to respond using the mouse and analysed mouse trajectory, in this study, we required subjects to digit their responses in an edit box, a condition which mimics online form completion. Lie detection via keystroke dynamics is more suitable than mouse tracking for online contexts (e.g. to verify the authenticity of information typed by the user during an online subscription). Moreover, this setting allows for the use of covert lie detection, a lie-detection procedure in which the respondent is unaware of being tested for lies.

The procedure proposed herein seems to have the following advantages:

- It is one of the few techniques that can be used to implement covert lie detection.
- It does not require external instrumentation because only a computer and a keyboard are required.
- The number of predictors is high, rendering the development of effective countermeasures to lie detection difficult.

We conducted a proof-of-concept lie-detection experiment in which liars (namely lying about their personal identity) are identified based on their anomalous typing style (i.e. keystroke dynamics). Questions about identity that were expected were mixed with unexpected questions. This approach confirmed that unexpected questions effectively increase liars’ cognitive and facilitate their differentiation from truth tellers. The predictor that most contributed to this classification was the number of errors made by liars, mainly when presented with these unexpected questions about identity. Furthermore, liars exhibited longer reaction times between the onset of the question and the start of their response in the edit box. This result shows that liars require more time to retrieve their response. The increase in time was not only limited to the onset of the response but was also observed in the time required to type the full response and in the time taken to confirm the response after the last letter was pressed. Liars take more time to verify their untruthful responses with respect to truth tellers offering true responses. In short, final rechecking of the response took more time for liars than truth tellers. Examples of the prototypical deceptive and truthful keystroke patterns are reported in Table 8, which is useful for visualising the difference between liars and truth tellers revealed by keystroke pattern analysis.

**Table 8:** Table reports the prototypical keystroke pattern of a liar and a truthteller for the 5 predictors used in the classification models.

Feature	Prototypical truthteller	Prototypical liar
Number of errors	0/18 = 0.00	7/18 = 0.39
Prompted-firstdigit adjusted GULPEASE	1649 ms	3508 ms
Firstdigit-enter	3123 ms	3567 ms
Writing time	281 ms	442 ms
Time key before enter down	462 ms	739 ms

The number of errors is defined as the number of fields compiled by entering the wrong information. The prompted-firstdigit adjusted GULPEASE, is the interval between the onset of the sentence on the computer screen and the first key pressed. The firstdigit-enter is the time between the press of the first key and the press of ENTER. The writing time corresponds to the firstdigit-enter divided by the number of characters typed. The time key before enter down is the time between the press of the last key and the press of ENTER.

To conclude, the technique of asking unexpected questions combined with keystroke pattern analysis may be an efficient instrument to spot fake identities with the possibility of easy application in the online context. The methodology that we proposed in this paper showed an accuracy similar to that obtained in other studies using the unexpected-questions technique^11^ as well as other RT-based lie-detection techniques^21,22^. However, it is suitable for a broader range of applications, such as the web deception.

As anticipated in the previous section, the resistance to countermeasures was not directly tested in the present paper; it remains an open issue. The high number of predictors leads one to think that it is possible to develop many models that take into account different features for the prediction. This makes it more difficult for the user to fully control the lie-detection machine via efficient, planned countermeasures. However, our study also shows that some features, such as the number of errors, play an important role in classification accuracy. To point out this issue, we plan to specifically test the technique’s resistance to countermeasures by instructing subjects to beat the lie-detection machine with explicit strategies.

Before the experimental task, the subjects spent a few minutes learning the fake identity. In some real cases (e.g. the terrorist traveling with a false passport), the subject spent more time learning more the false identity in greater depth. However, it is also true that in other real cases (e.g. the user trying to subscribe to a website with a fake identity), people are not so well prepared. Since the method is based on asking unexpected questions, the time taken to learn the information is not crucial. Indeed, what is crucial to beat the test is to be prepared to respond with unexpected information. Future experiments will be directed at testing the accuracy of the method in the case of a subject who is aware of the underlying logic of the task in advance or who knows the possible unexpected questions in advance. Asking unexpected questions entails a complex crafting process that requires identifying what would be unexpected questions for the examinee. If an unexpected question is not really unexpected (as the subject may prepare him or herself in advance), it becomes an expected question and loses its efficacy. Though unexpected questions can be varied in content (e.g. postal code), another future direction could be to tune new strategies, different from unexpected questions, to increase liars’ cognitive load, thereby resulting in an alteration of keystroke dynamics.

Other limits of the present investigations include the following:

- The experiment was conducted using students; hence, a replication with older and less-skilled subjects is required, for age and writing skills may mediate the effect of lying.
- Some of the unexpected questions included in the task rely on information that cannot be generalised to all cultures and ethnicities. An example is the unexpected question about the zodiac sign, which may be well-known personal information in some culture but not in others.

In line with these limitations, future studies will address the collection of additional data from populations with different ages, skills and cultures.

## Electronic supplementary material

Supplementary Tables
