# Supplementary material for: Covert lie detection using keyboard dynamics
Source: Sci Rep. 2018 Jan 31;8:1976. doi: 10.1038/s41598-018-20462-6 (PMC5792443; doi:10.1038/s41598-018-20462-6)
Supplement: Supplementary file 1 — Supplementary Tables [file 41598_2018_20462_MOESM1_ESM.pdf]

Covert lie detection using keyboard dynamics

Merylin Monaro[1], Chiara Galante[2], Riccardo Spolaor[1], Qian Qian Li[1], Luciano Gamberini[2,3], Mauro Conti[3,4] and Giuseppe Sartori[2,3] *

[1] PhD Program in Brain, Mind and Computer Science, University of Padova, 35122, Italy
[2] University of Padova, Department of General Psychology, Padova, 35131, Italy
[3] University of Padova, Human Inspired Technology Research Centre, Padova, 35122, Italy
[4] University of Padova, Department of Mathematics, Padova, 35131, Italy
*giuseppe.sartori@unipd.it

Supplementary Information

Table S1. Complete list of the 62 attributes extracted for each subject's typed response.

Number of errors
Prompted-firstdigit
Prompted-firstdigit adjusted GULPEASE
Prompted-enter
Firstdigit-enter
Time before enter key down
Time before enter key flight
Answer length
Writing time
Di-graph down time average
Di-graph down time maximum
Di-graph down time minimum
Di-graph down time median
Di-graph down time standard deviation
Di-graph down time variance
Di-graph up time average
Di-graph up time maximum
Di-graph up time minimum
Di-graph up time median
Di-graph up time standard deviation
Di-graph up time variance
Di-graph up and down time average
Di-graph up and down time maximum
Di-graph up and down time minimum
Di-graph up and down time median
Di-graph up and down time standard deviation
Di-graph up and down time variance
Di-graph press time average
Di-graph press time maximum
Di-graph press time minimum
Di-graph press time median
Di-graph press time standard deviation
Di-graph press time variance
Di-graph flight time average
Di-graph flight time maximum
Di-graph flight time minimum
Di-graph flight time median
Di-graph flight time standard deviation
Di-graph flight time variance
Tri-graph down time average
Tri -graph down time maximum
Tri -graph down time minimum
Tri -graph down time median
Tri -graph down time standard deviation
Tri -graph down time variance

Tri -graph up time average
Tri -graph up time maximum
Tri -graph up time minimum
Tri -graph up time median
Tri -graph up time standard deviation
Tri -graph up time variance
Tri -graph up and down time average
Tri -graph up and down time maximum
Tri -graph up and down time minimum
Tri -graph up and down time median
Tri -graph up and down time standard deviation
Tri -graph up and down time variance
Number of Shift
Number of Del
Number of Canc
Number of Space
Number of Arrows

Table S2. Classification accuracies and metrics obtained by eliminating one by one the five final predictors (errors, prompted-firstdigit adjusted GULPEASE, firstdigit-enter, writing time, time key before enter down).

Results obtained by eliminating errors:

**10-fold cross-validation**								
**Classifier**	**Accuracy**	**TP Rate**	**FP Rate**	**Precision**	**Recall**	**F-Measure**	**ROC Area**	**PRC Area**
Logistic	82.5%	0.825	0.175	0.826	0.825	0.825	0.865	0.852
SVM (SMO)	82.5%	0.825	0.175	0.832	0.825	0.824	0.825	0.771
LMT	82.5%	0.825	0.175	0.826	0.825	0.825	0.915	0.913
Random Forest	82.5%	0.825	0.175	0.826	0.825	0.825	0.834	0.794
**Test**								
**Classifier**	**Accuracy**	**TP Rate**	**FP Rate**	**Precision**	**Recall**	**F-Measure**	**ROC Area**	**PRC Area**
Logistic	65%	0.650	0.350	0.700	0.650	0.627	0.850	0.865
SVM (SMO)	65%	0.650	0.350	0.700	0.650	0.627	0.650	0.605
LMT	65%	0.650	0.350	0.700	0.650	0.627	0.840	0.81
Random Forest	70%	0.700	0.300	0.730	0.700	0.688	0.865	0.864

Results obtained by eliminating prompted-firstdigit adjusted GULPEASE:

**10-fold cross-validation**								
**Classifier**	**Accuracy**	**TP Rate**	**FP Rate**	**Precision**	**Recall**	**F-Measure**	**ROC Area**	**PRC Area**
Logistic	92.5%	0.925	0.075	0.926	0.925	0.925	0.967	0.957
SVM (SMO)	92.5%	0.925	0.075	0.935	0.925	0.925	0.925	0.897
LMT	92.5%	0.925	0.075	0.935	0.925	0.925	0.993	0.993

Random Forest	95%	0.950	0.050	0.950	0.950	0.950	0.972	0.972
**Test**								
**Classifier**	**Accuracy**	**TP Rate**	**FP Rate**	**Precision**	**Recall**	**F-Measure**	**ROC Area**	**PRC Area**
Logistic	90%	0.900	0.100	0.917	0.900	0.899	0.970	0.950
SVM (SMO)	90%	0.900	0.100	0.917	0.900	0.899	0.900	0.867
LMT	95%	0.950	0.050	0.955	0.950	0.950	1.000	1.000
Random Forest	100%	1.000	0.000	1.000	1.000	1.000	1.000	1.000

Results obtained by eliminating firstdigit-enter:

**10-fold cross-validation**								
**Classifier**	**Accuracy**	**TP Rate**	**FP Rate**	**Precision**	**Recall**	**F-Measure**	**ROC Area**	**PRC Area**
Logistic	92.5%	0.925	0.075	0.926	0.925	0.925	0.964	0.953
SVM (SMO)	95%	0.950	0.050	0.950	0.950	0.950	0.950	0.928
LMT	97.5%	0.975	0.025	0.976	0.975	0.975	1.000	1.000
Random Forest	92.5%	0.925	0.075	0.926	0.925	0.925	0.974	0.974
**Test**								
**Classifier**	**Accuracy**	**TP Rate**	**FP Rate**	**Precision**	**Recall**	**F-Measure**	**ROC Area**	**PRC Area**
Logistic	100%	1.000	0.000	1.000	1.000	1.000	1.000	1.000
SVM (SMO)	95%	0.950	0.050	0.950	0.950	0.950	0.950	0.928
LMT	90%	0.900	0.100	0.917	0.900	0.899	1.000	1.000
Random Forest	95%	0.950	0.050	0.955	0.950	0.950	0.990	0.991

Results obtained by eliminating writing time:

**10-fold cross-validation**								
**Classifier**	**Accuracy**	**TP Rate**	**FP Rate**	**Precision**	**Recall**	**F-Measure**	**ROC Area**	**PRC Area**
Logistic	92.5%	0.925	0.075	0.926	0.925	0.925	0.951	0.947
SVM (SMO)	95%	0.950	0.050	0.950	0.950	0.950	0.950	0.928
LMT	97.5%	0.975	0.025	0.976	0.975	0.975	1.000	1.000
Random Forest	90%	0.900	0.100	0.900	0.900	0.900	0.974	0.975
**Test**								
**Classifier**	**Accuracy**	**TP Rate**	**FP Rate**	**Precision**	**Recall**	**F-Measure**	**ROC Area**	**PRC Area**
Logistic	100%	1.000	0.000	1.000	1.000	1.000	1.000	1.000

SVM (SMO)	90%	0.900	0.100	0.917	0.900	0.899	0.900	0.867
LMT	90%	0.900	0.100	0.917	0.900	0.899	1.000	1.000
Random Forest	95%	0.950	0.050	0.955	0.950	0.950	1.000	1.000

Results obtained by eliminating time before enter key down:

**10-fold cross-validation**								
**Classifier**	**Accuracy**	**TP Rate**	**FP Rate**	**Precision**	**Recall**	**F-Measure**	**ROC Area**	**PRC Area**
Logistic	90%	0.900	0.100	0.900	0.900	0.900	0.980	0.982
SVM (SMO)	95%	0.950	0.050	0.950	0.950	0.950	0.950	0.928
LMT	97.5%	0.975	0.025	0.976	0.975	0.975	1.000	1.000
Random Forest	90%	0.900	0.100	0.900	0.900	0.900	0.976	0.977
**Test**								
**Classifier**	**Accuracy**	**TP Rate**	**FP Rate**	**Precision**	**Recall**	**F-Measure**	**ROC Area**	**PRC Area**
Logistic	90%	0.900	0.100	0.917	0.900	0.899	0.980	0.982
SVM (SMO)	90%	0.900	0.100	0.917	0.900	0.899	0.900	0.867
LMT	90%	0.900	0.100	0.917	0.900	0.899	1.000	1.000
Random Forest	95%	0.950	0.050	0.955	0.950	0.950	0.990	0.991

Table S3. Classification accuracies and metrics obtained by analysing only control questions.

**10-fold cross-validation**								
**Classifier**	**Accuracy**	**TP Rate**	**FP Rate**	**Precision**	**Recall**	**F-Measure**	**ROC Area**	**PRC Area**
Logistic	40%	0.400	0.600	0.400	0.400	0.400	0.303	0.398
SVM (SMO)	45%	0.450	0.550	0.448	0.450	0.444	0.450	0.478
LMT	45%	0.450	0.550	0.237	0.450	0.310	0.405	0.476
Random Forest	55%	0.550	0.450	0.550	0.550	0.550	0.530	0.538
**Test**								
**Classifier**	**Accuracy**	**TP Rate**	**FP Rate**	**Precision**	**Recall**	**F-Measure**	**ROC Area**	**PRC Area**
Logistic	60%	0.600	0.400	0.619	0.600	0.538	0.630	0.627
SVM (SMO)	45%	0.450	0.550	0.402	0.450	0.373	0.450	0.480
LMT	50%	0.500	0.500	0.250	0.500	0.333	0.500	0.500
Random Forest	40%	0.400	0.600	0.396	0.400	0.394	0.390	0.494

Table S4. Classification accuracies and metrics obtained using a new set of predictors (errors, prompted-firstdigit, prompted-enter, time before enter key flight, di-graph down time average).

**10-fold cross-validation**								
**Classifier**	**Accuracy**	**TP Rate**	**FP Rate**	**Precision**	**Recall**	**F-Measure**	**ROC Area**	**PRC Area**
Logistic	92.5%	0.925	0.075	0.926	0.925	0.925	0.938	0.921
SVM (SMO)	95%	0.950	0.050	0.950	0.950	0.950	0.950	0.928
LMT	97.5%	0.975	0.025	0.976	0.975	0.975	1.000	1.000
Random Forest	90%	0.900	0.100	0.904	0.900	0.900	0.974	0.973
**Test**								
**Classifier**	**Accuracy**	**TP Rate**	**FP Rate**	**Precision**	**Recall**	**F-Measure**	**ROC Area**	**PRC Area**
Logistic	100%	1.000	0.000	1.000	1.000	1.000	1.000	1.000
SVM (SMO)	90%	0.900	0.100	0.917	0.900	0.899	0.900	0.867
LMT	90%	0.900	0.100	0.917	0.900	0.899	1.000	1.000
Random Forest	90%	0.900	0.100	0.917	0.900	0.899	1.000	1.000

Table S5. Classification accuracy and metrics obtained from the one-class classifier.

**Test**								
**Classifier**	**Accuracy**	**TP Rate**	**FP Rate**	**Precision**	**Recall**	**F-Measure**	**ROC Area**	**PRC Area**
One-class classifier	85%	0.850	0.150	0.885	0.850	0.847	1.000	1.000

Table S6. Classification accuracies and metrics obtained testing the new sample of 151 online-participants, using a new set of predictors (errors, prompted-firstdigit, prompted-enter, time before enter key flight, di-graph down time average).

**Test on 151 online-recruited participants**								
**Classifier**	**Accuracy**	**TP Rate**	**FP Rate**	**Precision**	**Recall**	**F-Measure**	**ROC Area**	**PRC Area**
Logistic	90.1%	0.901	0.098	0.902	0.901	0.901	0.958	0.947
SVM (SMO)	90.1%	0.901	0.083	0.911	0.901	0.901	0.909	0.871
LMT	90.7%	0.907	0.074	0.920	0.907	0.908	0.960	0.954
Random Forest	90%	0.900	0.100	0.904	0.900	0.900	0.974	0.973
